# A Report of a Child with SEC31A-Related Neurodevelopmental Disorder

**DOI:** 10.3390/ijms26115296

**Published:** 2025-05-30

**Authors:** Ruqaiah AlTassan, Hanan AlQudairy, Biam Saydo, Aseel Alammari, Kelly J. Cardona Londoño, Khushnooda Ramzan, Dilek Colak, Stefan T. Arold, Namik Kaya

**Affiliations:** 1 Department of Medical Genomics, Genomic Medicine Center of Excellence, King Faisal Specialist Hospital and Research Centre, Riyadh 11211, Saudi Arabia; raltassan@kfshrc.edu.sa; 2 College of Medicine, AlFaisal University, Riyadh 11533, Saudi Arabia; bsaydo@alfaisal.edu; 3 Translational Genomic Department, Genomic Medicine Center of Excellence, King Faisal Specialist Hospital and Research Centre, Riyadh 11211, Saudi Arabia; halqudairy@kfshrc.edu.sa (H.A.); aalammari1@kfshrc.edu.sa (A.A.); 4 KAUST Center of Excellence for Smart Health, Biological and Environmental Science and Engineering Division, King Abdullah University of Science and Technology, Thuwal 23955-6900, Saudi Arabia; kelly.cardonalondono@kaust.edu.sa; 5 Clinical Genomics Department, Genomic Medicine Center of Excellence, King Faisal Specialist Hospital and Research Centre, Riyadh 11211, Saudi Arabia; kramzan@kfshrc.edu.sa; 6 Molecular Oncology Department, King Faisal Specialist Hospital and Research Centre, Riyadh 11211, Saudi Arabia; dkcolak@gmail.com

**Keywords:** SEC31A, COPII, neurodevelopmental disorder, seizure disorder, corpus callosum hypogenesis

## Abstract

SEC31A-related neurodevelopmental disorder (Halperin–Birk syndrome) was recently identified in two siblings who shared the phenotype of profound developmental delay, structural brain defects, spastic quadriplegia with multiple contractures, seizures, dysmorphism, and optic nerve atrophy. Both patients died during childhood. In this study, we identified an additional patient who suffers from global developmental delay and seizures. Genetic analysis inclusive of whole exome and genome sequencing identified a homoallelic variant in the *SEC31A* (p.Cys453Trp). Various in silico classifiers predicted a deleterious effect of the replacement of cystein with tryptophan at the 453rd position. Protein–protein interaction (PPI) network analysis of SEC31A revealed high-confidence interactions with SEC13, SEC23A, and SEC23B, suggesting potential regulatory roles in these processes. Structural analysis of the SEC31A–SEC13 interaction and the Cys453Trp mutant in SEC31A predicted that the stability of coat protein complex II would be compromised. Our findings support the clinical correlation of *SEC31A* variants with neurodevelopmental disorder.

## 1. Introduction

SEC31A is abundantly and ubiquitously expressed in various tissues, and it is the functional mammalian homologue of the yeast Sec31 protein [1,2,3,4]. *SEC31A* is located on chromosome 4q21.22 and encodes one of the five subunits of the coat protein complex II (COPII), which is crucial for the cellular trafficking of proteins and lipids from the endoplasmic reticulum (ER) to the Golgi apparatus [1]. Given the critical role of COPII at the cellular level, mutations in its subunits have been associated with disorders that affect multiple systems, including the liver, immune system, and hematological functions [5]. Although the exact pathogenesis of COPII-related diseases remains unclear, mutations in nearly all COPII proteins, with the exception of SEC13A, have been identified in humans. Until recently, mutations in the *SEC31A* were not associated with any specific disorder. However, Halperin et al. (2019) described two siblings with a neurodevelopmental disorder characterized by structural brain defects, spastic quadriplegia with multiple contractures, profound developmental delay, seizures, and dysmorphism due to a null mutation in the *SEC31A* [OMIM 618651] [4]. More recently, another patient was reported with a splicing variant leading to exon skipping (exon 12) and an in-frame deletion of several amino acids [6].

In this study, we report the third homozygous missense variant in *SEC31A*, detected by whole exome sequencing (WES) and then by confirmatory whole genome sequencing (WGS), in a child presenting with global developmental delay, seizures, facial dysmorphism, and hearing impairment.

## 2. Results

### 2.1. Case Report

**Figure 1:**
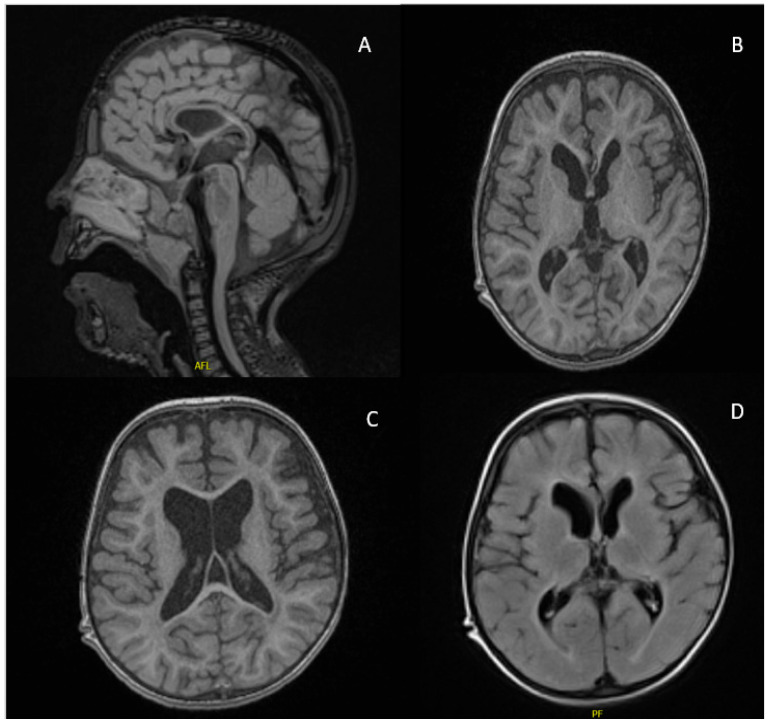
Brain MRI findings of the patient at the age of 3 years showing corpus callosum thinning ((**A**), T1), hypomyelination, and brain volume loss ((**B**,**C**) (T1), and (**D**) (T2)).

**Figure 2:**
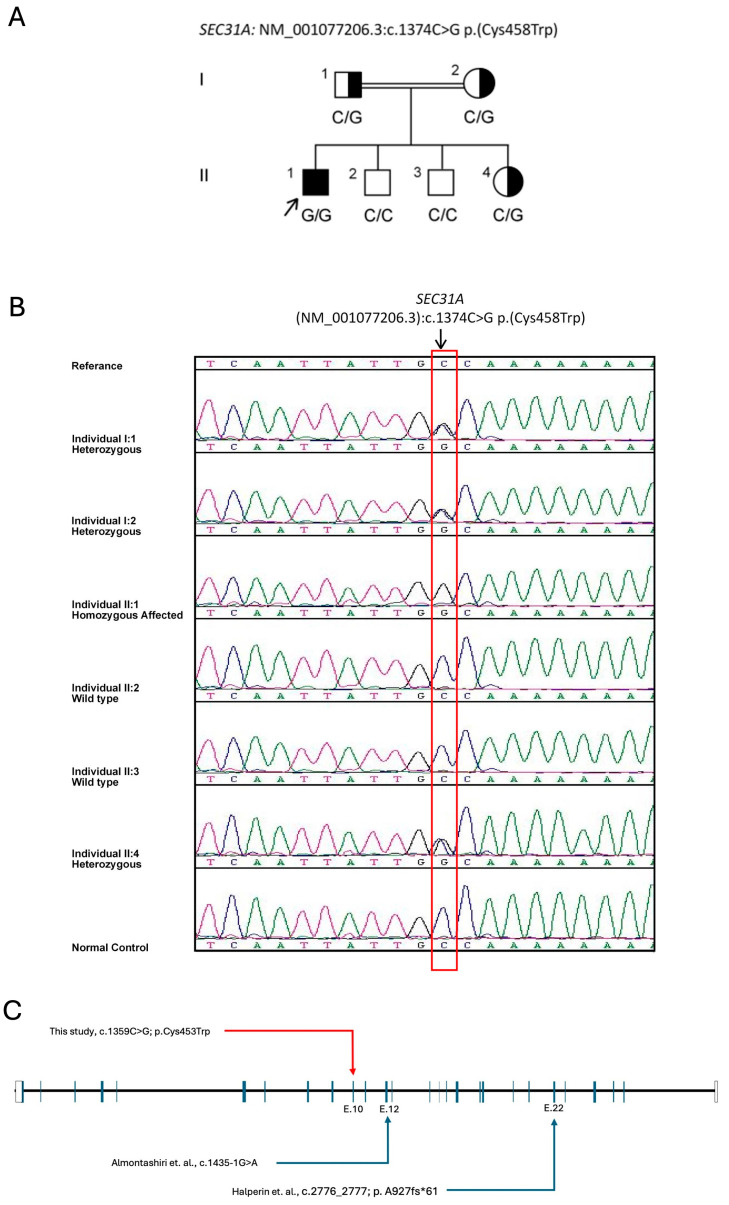
Genetic analysis of *SEC13* variant: (**A**) Segregation analysis of the *SEC31A* variant (c.1359C > G; p.Cys453Trp). (**B**) Studied family pedigree indicating the index patient (black arrow) and siblings (filled square refers to the male patient; squares: males; circles: females; genotypes are given below each symbol). (**C**) Schematic drawing of the reported *SEC31A* variants linked to Halperin–Birk syndrome (OMIM #610257; phenotype MIM: 618651) [4,6].

A 5-year-old male patient, born to consanguineous parents, was delivered at term via vaginal delivery with good antenatal and postnatal care. He presented with global developmental delay, seizure disorder, hypotonia, spasticity, dysphagia, dysmorphic features, and bilateral hearing loss. Concerns arose regarding delayed motor milestone acquisition at 12 months old, and the family sought medical evaluation at the age of 2 years. By age 3, he began experiencing seizures and was started on levetiracetam. Physical assessment at the age of 5 years revealed dysmorphic facial features, including a prominent nasal bridge, hypertelorism, epicanthal folds, frontal bossing, and prominent ears. Growth parameters indicated microcephaly, failure to thrive, and short stature. The patient exhibited central hypotonia and spasticity in both upper and lower limbs. Developmental assessment showed delays in cognition, motor function, and speech. He started to roll over and sit without support at the age of 4 years and, at the time of last assessment, he was wheel-chair dependent. He cannot say a single word. Audiology assessment indicated moderate to severe bilateral hearing loss, whereas his eye exam was normal. Brain MRI revealed hypogenesis of the corpus callosum, hypomyelination, and diffuse brain atrophy (Figure 1). His seizures remained under control with antiepileptic medication and he is enrolled in a comprehensive physiotherapy program. The patient also suffered from oropharyngeal dysphagia and recurrent aspiration, which slightly improved with the addition of thickeners to his formula feeding. WES performed on genomic DNA (extracted from peripheral blood of the patient) identified a novel homozygous variant in *SEC31A* (NM_001191049.2): c.1359C > G; (p.Cys453Trp)/chr4-83785575-G-C (hg19). The result was further confirmed by an in-house WGS to rule out any other contributing variant(s) to the patient’s phenotype. There was no other plausible candidate variant detected in both genomic assays. Parental carrier testing confirmed the in-trans inheritance (Figure 2A), and family segregation analysis (Figure 2B) in his three apparently healthy siblings supported the variant’s causality. The variant was detected in a heterozygous state in his 11-year-old sister and was absent in the other 15- and 13-year-old brothers.

### 2.2. The Variant and in Silico Pathogenesis Analysis

To further explore the potential impact of this novel variant, we utilized several well-established in silico prediction tools. These tools were selected based on their broad use in variant interpretation guidelines and their diverse methodological approaches, including conservation analysis, machine learning, and ensemble models. Tools such as SIFT [7], PolyPhen-2 [8], MutationTaster [9,10], and PROVEAN [11] were used to assess evolutionary conservation and amino acid changes, while others like ClinPred [12], MetaRNN [13], MutPred, and CADD were used to apply integrated models or deep learning trained on pathogenic and benign datasets. A summary of the prediction results is presented in Table 1, showing the scores alongside the predicted functional impact.

The majority of the tools consistently predicted the variant to be deleterious or disease associated, indicating strong computational evidence supporting a pathogenic effect (PP3).

### 2.3. Gene Network Analysis (GNA)

**Figure 3:**
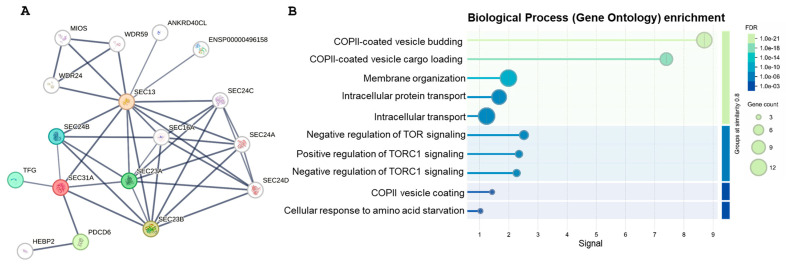
Protein–protein interaction (PPI) network analysis of SEC31: (**A**) STRING-generated PPI network showing SEC31A’s interactions with other proteins. Nodes represent proteins, and edges indicate high-confidence interactions (score > 0.7) supported by experimental evidence. Colored nodes denote direct interactors, while uncolored nodes represent indirect associations. The edge thickness indicates the confidence level, with thicker edges indicating stronger evidence (see Legend). (**B**) Gene ontology (GO) enrichment analysis. Significantly enriched biological processes identified using STRING’s functional annotation tool (adjusted *p* < 0.05) among the interacting proteins.

STRING analysis revealed a high-confidence protein–protein interaction (PPI) network for SEC31A, consisting of 17 nodes and 40 edges, based on experimentally validated interactions. The network displayed a core cluster of proteins closely associated with SEC31A, suggesting its involvement in functionally related pathways (Figure 3).

Biological process and pathway enrichment analysis identified significant enrichment in COPII-coated vesicle budding, protein processing in endoplasmic reticulum, and mTOR signaling pathways (adjusted *p* < 0.05). Notably, interactions with SEC13, SEC23A, and SEC23B suggest potential regulatory roles in these processes (Figure 3).

Among the key interactors, SEC13, SEC23A, and SEC23B—essential components of the COPII vesicle trafficking system—formed a central hub in the network. All interactions were supported by a confidence score > 0.7, indicating strong functional associations. SEC13 was identified as a direct interactor of SEC31A, consistent with its known role in forming the outer layer of the COPII coat. Additionally, SEC23A and SEC23B, which are part of the inner coat complex and act as GTPase-activating proteins (GAPs) for SAR1, also showed high-confidence interactions with SEC31A. This supports SEC31A’s critical role in vesicle budding and transport from the ER to the Golgi apparatus.

These findings underscore the critical role of SEC31 in intracellular trafficking and maintaining protein secretion pathways. Taken together, the STRING-based interaction network provides strong support for the established role of SEC31 in COPII vesicle formation, highlighting its direct interactions with SEC13, SEC23A, and SEC23B in coordinating cargo selection and vesicle budding at the ER membrane.

### 2.4. Structural Modeling of the Variant

**Figure 4:**
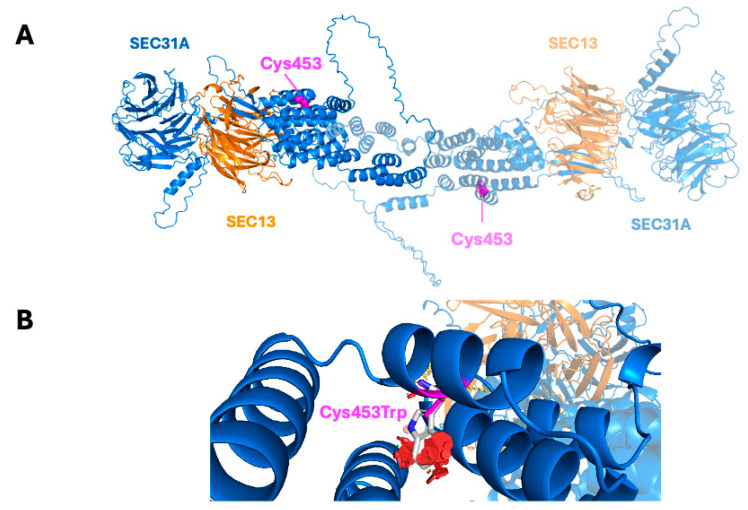
Three-dimensional structural model of the SEC31A–SEC13 interaction and the Cys453Trp mutant in SEC31A. (**A**) AlphaFold3 structural model of the dimeric complex between SEC31A isoform 7 (residues 1–810; (dark and light blue) and the canonical isoform of SEC13 (dark and light orange). The location of residue Cys453 in SEC31A is highlighted in magenta. (**B**) Close-up view of the molecular environment of the Cys453Trp variant in SEC31A. The wild-type cysteine side chain is depicted in magenta. The mutant tryptophan side chain is colored by elements, showing carbon in white and nitrogen in blue. Structural clashes introduced by the mutant are represented by red circles, with their orientation and diameter indicating the direction and severity of the clashes. The region surrounding residue Cys453 is predicted with high confidence (pLDDT between 70 and 90), supporting the reliability of the structural interpretation at the mutation site. However, the overall model confidence is moderate (pTM: 0.52; ipTM: 0.49), and interpretation of long-range or inter-subunit interactions might be cautious. Full confidence metrics, including residue-level pLDDT and predicted aligned error (PAE), are provided in Supplementary Figure S1.

The Cys453Trp mutation occurs in the α-solenoid domain of SEC31A. Cys453 plays a crucial role in the hydrophobic packing of this helical domain. Substituting Cys453 with the much larger tryptophan is predicted to cause steric clashes that weaken or disrupt the α-solenoid domain. The correct architecture and stability of the α-solenoid domain of SEC31A are essential for forming the native COPII framework, as they ensure the proper orientation and spacing of the interactions between the WD40 domains of SEC31A and SEC13. Therefore, the Cys453Trp variant is expected to hinder stable COPII formation (Figure 4A,B, Supplementary Figure S1).

Of note, the isoform analyzed (NM_001191049.2) differs from the canonical SEC31A isoform (Uniprot accession O94979-1) in two regions: the N-terminal sequence from positions 1 to 26 is replaced (length adjustment of −4 residues: MKLKEVDRTAMQAWSPAQNHPIYLAT → MLGESDERCTNAGSGCRRSSP), and residues 974 to 988 are missing (−15 residues). As a result, Cys453 corresponds to Cys458 in the canonical isoform.

### 2.5. ACMG Classification of c.1359C > G; p.Cys453Trp

Based on the current ACMG/AMP guidelines, the c.1359C > G would most probably be classified as likely pathogenic. This classification is supported by the variant’s segregation with disease in the family, extreme rarity across population databases including the CGMdb, Saudi Genome database, and other population specific databases; consistent pathogenic predictions from over 20 in silico pathogenicity prediction tools; a phenotype in the patient that matches previously reported cases; and supportive evidence from structural protein modeling and gene network analyses. Together (≥1 Strong + ≥1 Moderate + ≥1 Supporting, or ≥2 Moderate + ≥2 Supporting), these meet multiple moderate, supporting, and potentially strong criteria under the ACMG framework, justifying a likely pathogenic designation.

## 3. Discussion

COPII complex is composed of five different subunits assembled into an inner layer (SAR1, SEC23, and SEC24) surrounded by the outer layer (SEC13 and SEC31A) [30]. The main function of COPII is vesicles formation and protein transport from the ER to the ER–Golgi intermediate compartment (ERGIC) [31]. COPII has a critical role in many cellular processes including protein secretion and folding and overall cellular hemostasis [31]. Defects in COPII have been studied for various disease pathomechanisms including certain cancers, immune disorders, and congenital disorders of glycosylation [32]. Yet, its role in human diseases is still not well identified. In addition to SEC31A-related Halperin–Birk syndrome, four other Mendelian disorders have been linked to COPII subunits, including chylomicron retention disease due to allelic mutation in SAR1B, craniolenticulosutural dysplasia due to SEC23A mutations, congenital dyserythropoietic anemia type II secondary to SEC23B mutations, and autosomal recessive Cole–Carpenter syndrome 2, due to SEC24 complex defects [4,33,34,35]. The later one has been studied for its role in human glycosylation and recently linked to the expanded group of congenital disorders of glycosylation [36,37].

*SEC31A* encodes the outer layer of the coat protein complex II (COPII), which regulates the forward trafficking of protein and lipid payloads from the endoplasmic reticulum to the Golgi apparatus [38]. Disruption of the SEC31A leads to enhanced endoplasmic reticulum stress response and reduced cell viability [39].

Utilizing advanced genomic sequencing, we identified the fourth patient with a homoallelic missense variant in *SEC31A* and established its phenotype in comparison to the phenotypes resulting from the other two known variants (Table 2). The first description of a biallelic *SEC31A* null mutation in humans was by Halperin et al. (2019) [4]. He reported two siblings who had a profound neurological disease. They manifested the disease antenatally with intrauterine growth retardation, followed by postnatal developmental delay, spastic quadriplegia, pseudobulbar palsy, epilepsy, facial dysmorphism, neurosensory deafness, and optic nerve atrophy, and they passed away before the age of 4 years [5]. Very recently, another patient was reported with a lethal neonatal form of skeletal defects associated with other major congenital anomalies due to a *SEC31A* mutation [37].

Unlike previously reported patients, our family sought medical advice at the age of 12 months after noticing the patient’s failure to reach his developmental milestone. The clinical features observed in our patient lie within the same hallmark presentations as the other patients, most prominently the global developmental delay, the epilepsy, the hypotonia, and the corpus callosum agenesis. However, our patient did not exhibit eye abnormalities nor skeletal dysplasia, as was seen in the patient reported by Almontashiri et al. [6]. Phenotypically, our patient exhibited a milder spectrum among reported patients. The relatively stable postnatal presentation observed in our patient could be attributed to the missense variant in comparison to both mutations reported by Halperin et al. (2019) and Almontashiri et al. (2024), which are loss of function mutations and lead to truncation of SEC31A [4,6]. Moreover, other contributing factors that could lessen the phenotype severity in our patient cannot be ruled out, i.e., modifier genes, environmental factors, or genetic background differences.

The Cys453Trp mutation affects the SEC31A protein, a crucial player in COPII vesicle coat assembly. Various in silico classifiers predicted a deleterious effect of the replacement of cysteine with tryptophan at the 453rd position. A structural in silico analysis proposes that the Cys453Trp variant affects the stability and architecture of the α-solenoid domain of SEC31A. Thus, the variant is likely to perturb native COPII particle structure.

The role of SEC31A mutation in neurodevelopmental function is not yet fully understood. Studies involving SEC31A knockdown in flies have demonstrated defective brain development and early lethality, highlighting the gene’s potential involvement in neurodevelopment [4]. Our study, along with previous reports, supports the SEC31A effect on human neurodevelopment. This conclusion is limited by the small number of cases. Therefore, further studies with larger cohorts are urgently needed to explore the role of SEC31A as well as the COPII complex in health and disease.

## 4. Materials and Methods

### 4.1. DNA Isolation, Polymerase Chain Reaction (PCR)

Upon signing the written consent forms, peripheral blood samples (5 mL) from the patient and family members were collected in EDTA tubes. DNA isolation was carried out using the Gentra^®^ Puregene DNA Purification Kit (Gentra Systems, Inc., Minneapolis, MN, USA) according to company’s protocols. DNA was measured using a NanoDrop ND-1000 Spectrophotometer (ThermoFisher, Waltham, MA, USA) and checked on 1% agarose gel for integrity. Using the Primer-3 Web-Tool, we designed forward and reverse primers targeting the *SEC31A* variant.

### 4.2. Whole Exome Sequencing (WES)

Using the manufacturer’s guidelines (Illumina Inc., San Diego, CA, USA), the patient’s DNA was fragmented, pooled, paired-ended, and sequenced on an Illumina platform (HiSeq2500 from Illumina Inc., San Diego, CA, USA) with an average on-target coverage of approximately 30×. Call generation was carried out by Illumina’s pipeline (DRAGEN). All indels (with exception of CGM-confirmed variants) were further analyzed and validated by Sanger sequencing. All sequence changes were analyzed and filtered based on the previously established protocols and in-house pipelines that were developed for both research and clinical settings [40,41,42,43,44,45,46,47]. The DRAGEN Copy Number Variant (CNV) pipeline calls were utilized to discover likely CNV changes based on the Illumina-generated calls.

### 4.3. Whole Genome Sequencing (WGS)

Whole genome sequencing of the patient was carried out to confirm WES findings. WGS was performed using the TruSeq DNA prep kit, PCR-free library preparation approach from Illumina. Subsequent sequencing was carried out on a NovaSeq 6000 sequencing instrument (Illumina) to a sequencing depth of 30× median coverage.

### 4.4. Confirmatory Sanger Sequencing

Sequence changes in this individual were generated and then compared to the other available family members and population databases. Then, the selected variants were validated by Sanger sequencing according to standard protocols and based upon established quality metrics using fragments amplified by PCR. The sequencing results were analyzed using SeqMan Pro 15 (DNASTAR Inc., Madison, WI, USA).

### 4.5. Computational Structural Analysis of Mutants

The amino acid sequences of SEC31A and SEC13 were retrieved from the UniProt database (UniProt IDs: O94979-9 and P55735-1, respectively). A three-dimensional structural model of the SEC31A–SEC13 dimer was generated using the AlphaFold3 web server [48]. The predicted template-modeling scores (pTM) and interface-predicted template-modeling scores (ipTM) were = 0.52 and 0.49, respectively. The residue-specific predicted local distance difference tests (pLDDT) and the predicted aligned errors (PAE) for residue pairs are given in Supplementary Figure S1. To construct the dimeric complex, two subunits of SEC31A isoform 7 (residues 1–810) and one subunit of the canonical SEC13 were modeled in complex. For visualization purposes, a second unit of SEC13 was added to resemble the complex organization of the crystal structure of the yeast homolog sec13/31 (PDB ID: 2PM7). The structural model was manually inspected, and the Cys453Trp mutation in SEC31A was analyzed using PyMOL version 2.4.0 following the protocols presented in [49].

### 4.6. Gene Network Analysis

Protein–protein interaction (PPI) analysis for SEC31A was performed using STRING v12.0 [50]. Gene ontology (GO) enrichment analysis was performed using STRING’s functional annotation tools to identify biological processes significantly enriched (adjusted *p*-value < 0.05) among the interacting proteins. The final interaction network was visualized and analyzed using the STRING web interface.

## 5. Conclusions

In conclusion, we provide additional clinical report that a homozygous variant in SEC31A led to severe neurodevelopmental disorder characterized by global developmental delay, FTT, epilepsy, hypotonia, and hearing loss. Our study makes a valuable contribution to the understanding of SEC31A-related neurodevelopmental disorders by reporting the third case and demonstrating phenotypic variability. Future functional studies that are beyond the scope of this case report are needed to definitively establish pathogenicity of the variants in *SEC31A*.

## Figures and Tables

**Table 1:** In silico predictions of variant pathogenicity.

Prediction Tool	Score/Result	Pathogenicity Threshold	Interpretation *	Reference
AlphaMissense	0.9915	>0.564	Pathogenic (top 5% of pathogenic variants, rankscore = 0.95066)	[14]
BayesDel_addAF	0.3517	>0.16 = Pathogenic	Pathogenic (supports deleteriousness)	[15]
BayesDel_noAF	0.2674 (D)	>0.16 = Pathogenic	Pathogenic (independent of allele frequency)	[15]
CADD	21.6	>20	Pathogenic (top 1% of deleterious variants genome-wide)	[16]
ClinPred	0.9998	>0.5 = Pathogenic	Pathogenic (near-maximal confidence)	[12]
DANN	0.992	>0.95	Pathogenic (high-confidence prediction)	[17]
DEOGEN2	0.65427	0.89546	Deleterious prediction	[18]
ESM1b/Variped	−18.109	Lower = Deleterious	Deleterious (rankscore = 0.9965, “D” prediction)	[19]
Fathmm MKL	0.9551	>0.5 = Deleterious	Deleterious (group AEFGBI: likely functional impact)	[20]
Fathmm XF	0.8938	>0.5 = Deleterious	Deleterious (high confidence)	[21]
LRT	0	<0.05 = Deleterious	Deleterious (low conservation tolerance)	[22]
M_CAP	0.1809	>0.025 = Pathogenic	Pathogenic (moderate support)	[23]
MetaRNN	0.9436	>0.5 = Pathogenic	Pathogenic (high confidence)	[13]
MutPred	0.767	>0.5	Pathogenic (rankscore = 0.89452; gain of MoRF binding, *p* = 0.0355)	[24]
MutationTaster	1.0	N/A (qualitative)	Disease-Causing (simple_aae model)	[9]
MutationAssessor	3.575	>3.5 = High Impact	High Impact (functional hotspot)	[25]
Polyphen2 HDIV	1.0	>0.85 = Probably Damaging	Damaging (maximal confidence)	[8]
Polyphen2 HVAR	1.0	>0.85 = Probably Damaging	Damaging (consistent with HDIV)	[8]
PROVEAN	−10.38	≤−2.5 = Deleterious	Deleterious (far below threshold)	[26]
REVEL	0.498	>0.5	Uncertain significance (at border)	[27]
SIFT	0	<0.05 = Deleterious	Deleterious (strong evolutionary disruption)	[28]
SIFT4G	0	<0.05 = Deleterious	Deleterious (matches SIFT’s prediction)	[29]

* Out of 22 in silico classifiers, 21 predicted a pathogenic/deleterious outcome. Among the tested algorithms, only REVEL yielded a borderline score.

**Table 2:** Clinical features of the patient with SEC31A-related neurodevelopmental disorder.

		This Study	Almontashiri et al. (2024) [6]	Halperin et al. (2019) [4]	
Family		1	1	1	1
Subjects		1	1	1	2
Gender		Male	Female	Female	Male
Age at presentation		12 months	Antenatally	Birth	Birth
Age/outcome		5 years (alive)	15 days (died)	4 years (died)	2 years (died)
Consanguinity		(+)	(+)	(+)	(+)
Ethnicity		Arab	Arab	Middle east Bedouin	
Variant	c.DNA change	c.1359C > G	c.1435−1G > A	c.2776_2777 TA duplication	
	Amino acid change	p.Cys453Trp	-	p.A927fs*61	
	Zygosity	Homozygous	Homozygous	Homozygous	
Neurological findings		Seizures (generalized tonic–clonic)	No seizure	Seizures (focal and generalized tonic–clonic)	
		Global developmental delay (cognition, motor, speech) Central hypotonia Hyperreflexia Spasticity	Hypotonia Hyperreflexia	Global developmental delay (cognition, motor, speech) Spastcic quadriplasia Hypotonia	
Neuroimaging		Corpus callosum hypogenesis Hypomyelination Diffuse brain atrophy	Interhemispheric cyst Absent corpus callosum	Semilobar holoprosencephaly Enlargement of the subarachnoid space Corpus callosum agenesis Ventriculomegaly Colpocephaly	
EEG		Abnormal electric activity	-	Disorganized background activity with an epileptic pattern of bilateral sharp waves and spikes discharges	
Growth parameters		Microcephaly Short stature	Microcephaly Short stature	Microcephaly	
Cardiac		Normal	Bradycardia	Peri-membranotic VSD	
Genitourinary		Normal	Normal	Normal	
GI problems		Constipation Dysphagia Recurrent aspiration Failure to thrive	Not reported	Pseudobulbar palsy Recurrent aspirations Congenital diaphragmatic hernia Umbilical and inguinal hernia Gastro-esophageal reflux Feeding difficulties Failure to thrive	
Ophthalmological		Esotropia	Not reported	Nystagmus Lack of ocular fixation Bilateral nuclear cataracts	
Dysmorphic features		Prominent nasal bridge, hypertelorism, epicanthal fold, frontal bossing, prominent ears	Wide anterior fontanelle, sloping forehead, hypertelorism, malformed ears, depressed nasal bridge, retrognathia, short neck Skeletal anomalies	Pointed triangular face, micrognathia and high-arched palate, thick lips, long eyelashes	
Hearing		Bilateral hearing loss	Not reported	Bilateral hearing loss	

## Data Availability

Data are contained within the article and Supplementary Materials.

## Supplementary Materials

The following supporting information can be downloaded at: https://www.mdpi.com/article/10.3390/ijms26115296/s1.
