# Supplementary material for: A Report of a Child with SEC31A-Related Neurodevelopmental Disorder
Source: Int J Mol Sci. 2025 May 30;26(11):5296. doi: 10.3390/ijms26115296 (PMC12155280; doi:10.3390/ijms26115296)
Supplement: Supplementary file 1 [file ijms-26-05296-s001.zip › ijms-3602089-supplementary.pdf]

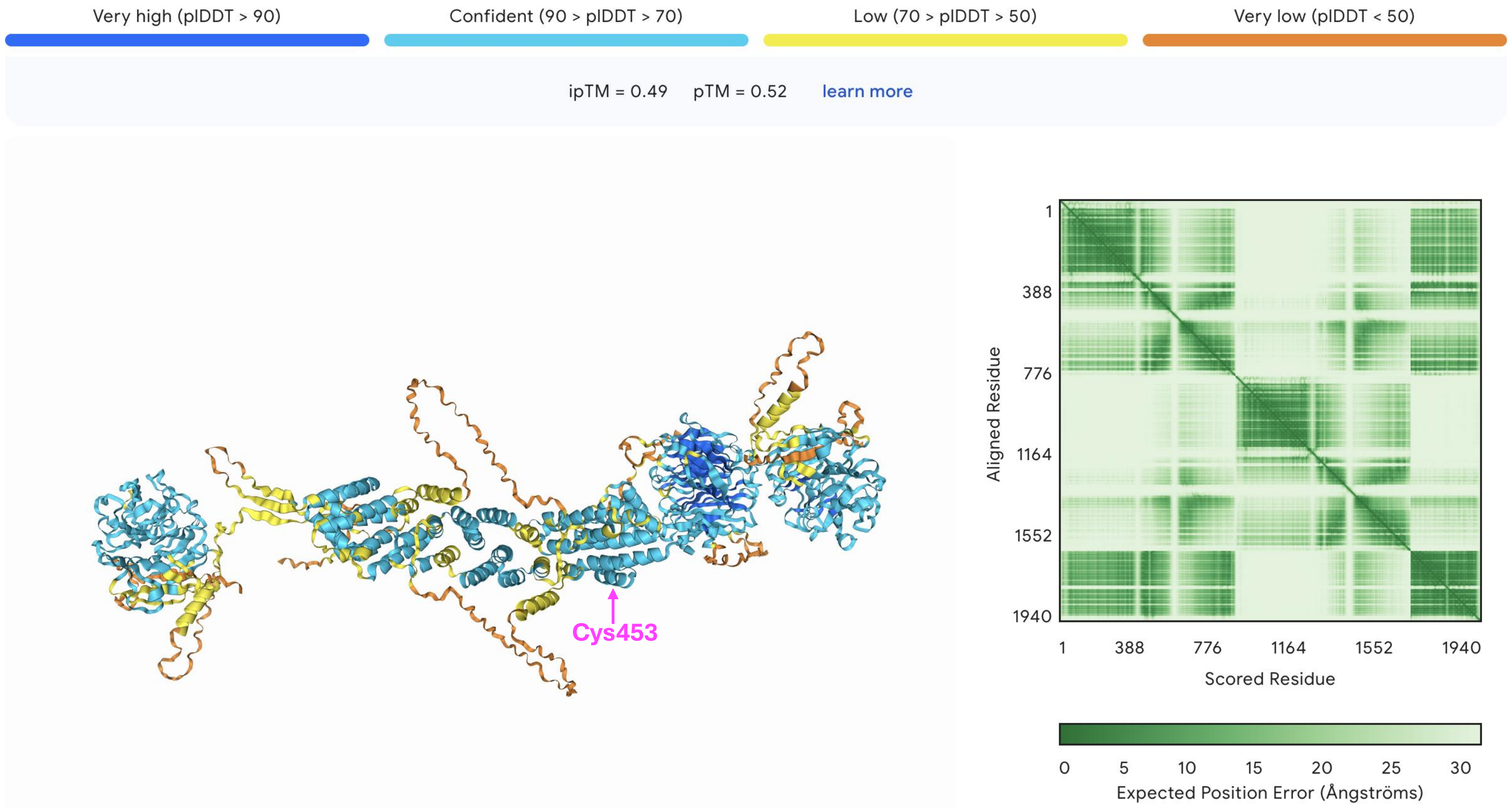

Supplementary Figure S1. Left panel. The AF3 Model of isoform 7 of SEC31A (residues 1-810) in complex with the canonical isoform of SEC13 (ipTM = 0.49, pTM = 0.52) residues are colored by confidence: dark blue - very high (pIDDT > 90), blue -high (90>pIDDT > 70), low – yellow (70 > pIDDT > 50), and very low – orange (plDDT < 50). Cys453 position is indicated with a magenta arrow. Right panel. Predicted alignment error (PAE) plot, the protein residues' positions are enumerated along the axes. In each square, the shade of green indicates error in Ångströms (Å) for each pair of residues. Dark green represents a good prediction (low error), whereas light green indicates poor prediction (high error).
